# Is city-level travel time by car associated with individual obesity or diabetes in Latin American cities? Evidence from 178 cities in the SALURBAL project

**DOI:** 10.1016/j.cities.2022.103899

**Published:** 2022-12

**Authors:** Xavier Delclòs-Alió, Daniel A. Rodríguez, Nancy López Olmedo, Carolina Pérez Ferrer, Kari Moore, Dalia Stern, Mariana Carvalho de Menezes, Letícia de Oliveira Cardoso, Xize Wang, Joanna M.N. Guimaraes, J. Jaime Miranda, Olga L. Sarmiento

**Affiliations:** a Institute of Urban and Regional Development, University of California, Berkeley, CA, USA; b Research Group on Territorial Analysis and Tourism Studies (GRATET), Department of Geography, Universitat Rovira i Virgili, Spain; c Department of City and Regional Planning & Institute for Transportation Studies, University of California, Berkeley, 228 Wurster Hall, Berkeley, CA 94720, USA; d Instituto Nacional de Salud Pública, Mexico, Avenida Universidad 655, 62100 Cuernavaca, Morelos, Mexico; e CONACyT-Instituto Nacional de Salud Pública, Cerrada de Fray Pedro de Gante 50, 14080 Mexico City, Mexico; f Dornsife School of Public Health, Drexel University, 3600 Market Street, Philadelphia, PA 19104, USA; g Department of Clinical and Social Nutrition, Federal University of Ouro Preto, Av. Pres. Antônio Carlos, 6627, Belo Horizonte 31270-901, Minas Gerais, Brazil; h Oswaldo Cruz Foundation, National School of Public Health, Av. Brasil 4365, Rio de Janeiro, 21040-900, Rio de Janeiro, Brazil; i Department of Real Estate, National University of Singapore, 4 Architecture Dr, 117566, Singapore; j CRONICAS Centre of Excellence in Chronic Diseases, Universidad Peruana Cayetano Heredia, Av. Armendariz 445, 15074 Lima, Peru; k School of Medicine, Universidad de Los Andes, Carrera 1, 111711 Bogotá, Colombia

**Keywords:** Car use, Travel time, Congestion, Obesity, Diabetes, Latin America

## Abstract

There is growing evidence that longer travel time by private car poses physical and mental risks. Individual-level obesity and diabetes, two of the main public health challenges in low- and middle-income contexts, could be associated to city-level travel times by car. We used individual obesity and diabetes data from national health surveys from individuals in 178 Latin American cities, compiled and harmonized by the SALURBAL project. We calculated city-level travel times by car using the Google Maps Distance Matrix API. We estimated associations between peak hour city-level travel time by car and obesity and diabetes using multilevel logistic regression models, while adjusting for individual characteristics and other city-level covariates. In our study we did not observe a relationship between city-level peak-hour travel time by car and individual obesity and diabetes, as reported in previous research for individual time spent in vehicles in high-income settings. Our results suggest that this relationship may be more complex in Latin America compared to other settings, especially considering that cities in the region are characterized by high degrees of population density and compactness and by a higher prevalence of walking and public transportation use.

## 1 Introduction

The use of private cars continues to impose enormous economic, environmental, and health costs around the world (Chapman, 2007; Gössling et al., 2019; Mueller et al., 2017; Shoup, 2015). This is especially worrisome in Latin American cities, where urbanization processes continue in tandem with unprecedented increases in passenger vehicle motorization rates (Inostroza et al., 2013), even considering that public transportation use is still very common (Ferrari et al., 2020). Higher levels of private car use results in increasing congestion levels and the consequent lengthening of overall daily travel times. In Bogotá, for example, automobile drivers lost 272 h to congestion in 2018, which is higher than any other city in the world (INRIX, 2019). Longer travel times by car not only imply significant financial burdens for individuals (Akbar and Duranton, 2017), but also pose relevant environmental impacts derived from the prolonged presence of cars, which result in increased emissions of pollutants and particulate matter, higher levels of energy consumption and related problems such as noise pollution (Rodrigue et al., 2013).

In high income countries, long travel times by car have been also associated with unhealthy lifestyles and poor health outcomes at the individual level. The main associations have been related to higher levels of physical inactivity, independent of leisure-time physical activity. This association should be particularly examined in Latin America, where urbanization rates, car use and the prevalence of chronic diseases related to sedentarism are particularly high (Webber et al., 2012). While previous studies in the Global North have generally linked the suburbanization process experienced throughout the twentieth century with lower population densities, higher levels of urban sprawl, higher car use and a subsequent increase in sedentarism and related health issues (Ewing et al., 2008; Ewing et al., 2014), these associations may be more complex in low- and middle-income countries. The urbanization process in regions such as Latin America have also experienced rapid growth and higher levels of fragmentation, but urban areas have generally remained dense and mixed-use (Inostroza et al., 2013). In turn, while the growth of urban areas may have increased car use and overall daily travel times, at the same time the nature of Latin American cities has also allowed for high levels of public transport ridership and use of active modes such as walking (Delclòs-Alió et al., 2021; Ferrari et al., 2020).

Considering the increasing relevance of obesity and diabetes as two of the main public health challenges in Latin America (Heisler et al.,2016; Jaacks et al., 2019; NCD Risk Factor Collaboration (NCD-RisC) - Americas Working Group, 2020; Popkin and Reardon, 2018; Werneck et al., 2019) and the limited evidence of their relationship with travel time by car in this geographic context, this paper aims to examine associations between citywide travel time and the risk of obesity and diabetes in a large selection of Latin American cities. This suggests that average longer travel times at the city-level may be detrimental to the health not only of individuals in vehicles, but also to the health of other city dwellers. The paper proposes two research questions: Is city-level peak-hour travel time associated with individual odds of obesity among residents of Latin American cities? Is city-level peak-hour travel time associated with individual odds of diabetes among residents of Latin American cities?

## 2 Literature review

In 2006, an ecological analysis in California found that commuters with the highest distance and time traveled also had the highest rates of physical inactivity and obesity (López-Zetina et al., 2006). Similarly, longer travel distance and time have been associated with higher BMI (body mass index) and waist circumference (Frank et al., 2004; Hoehner et al., 2012). In fact, a review showed that more car use was significantly associated with higher weight status in eight out of 10 studies (McCormack and Virk, 2014). In this line, a more recent review pointed to additional evidence supporting detrimental associations of prolonged car use and obesity (Sugiyama et al., 2020), specifically reporting that six out of six studies examining relationships of car use duration with obesity-related outcomes reported significant associations. Longitudinal studies also found that frequent and longer car use was associated with greater weight gain and higher cardiovascular mortality (Sugiyama et al., 2013; Warren et al., 2011).

Ecologically, a study found that commute mode diversity at the county level was associated with less obesity (Frederick et al., 2018). Other studies have also found similar associations between car use and outcomes such as diabetes and cardiovascular disease. A US cohort study found that participants with >10 h/week riding in a car had 50 % greater cardiovascular mortality than those who reported <4 h/week (Warren et al., 2011). Similarly, compared to spending 15 min/day or less in a car, spending >1 h/day was associated with higher BMI, waist circumference, fasting plasma glucose, and clustered cardio-metabolic risk (Sugiyama et al., 2016). In terms of diabetes, a recent study in Lisbon presented evidence supporting a negative association between active travel and hospital admissions for diabetes (Pereira et al., 2020), while a large-scale study focused in the US found different associations between transport-related physical activity and diabetes prevalence across race/ethnicity (Divney et al., 2019).

Fewer studies to date, however, have examined the association between travel time by car and health outcomes in low- and middle-income contexts (Anderson et al., 2019; Wang, Rodriguez, et al., 2019). Also, most studies such geographic contexts have either examined car-related factors and health outcomes related to sedentarism have focused on specific countries and have not focused on travel time. For example, a the recent study led by Patil and Sharma (2021), found that commuting by private modes in Navi Mumbai (India) was associated with getting overweight/obese. Similarly, a study set in China found that the association between neighbourhood characteristics and individual risk of being overweight among middle-aged and older adults was mediated by the odds of owning a car and spending less time being physically active (Wang, Feng, et al., 2019). Still in the Asian context, a study based in India found that motor vehicle ownership was significantly associated with obesity, particularly for women (Kellstedt et al., 2021), while a study set on rural China found that increases in population density were associated with weight gain through increasing car ownership (Yin et al., 2022).

In the Latin American context in particular, a study among Colombian men found that household motor vehicle ownership was associated with overweight, obesity and abdominal obesity (Parra et al., 2009). Another study in Chile found a positive association between active commuting and lower risk of obesity, diabetes and metabolic syndrome (Garrido-Méndez et al., 2017; Steell et al., 2018). Similarly, in a study focused on Peruvian rural-to-urban migrants it was hypothesized that daily habits characteristics from urban environments such as commuting might explain significant prevalence of obesity rates among these individuals (Carrillo-Larco et al., 2016). However, in other studies no significant associations have been found for commuting mode (car, public transport, active commuters) and obesity (Celis-Morales et al., 2019).

Beyond physical inactivity and sedentariness, there are other pathways that explain why city-level travel times by car may impact individual-level chronic diseases such as obesity and diabetes. For instance, higher exposure to air pollution generally produced by the prolonged presence of cars is associated with higher risk of obesity and diabetes (Alderete et al., 2018; Jerrett et al., 2014; Jerrett et al., 2017; Renzi et al., 2018; Weichenthal et al., 2014). Similarly, exposure to noise derived from traffic has also been linked to both obesity (Oftedal et al., 2015; Pyko et al., 2015) and diabetes (Clark et al., 2017; Dendup et al., 2018; Roswall et al., 2018). Lastly, although less studied, time spent traveling also impinges on time available for other activities like recreation and cooking healthy meals, both of which are likely to influence health outcomes (Christian, 2012; Euler et al., 2019).

All this considered, in this study we aim to explore the relationship between travel time at the city level and the risk of obesity and diabetes in large Latin American cities. However, our study not only aims to be of interest specifically for this geographic context, but also aims to contribute to a much-needed discussion regarding the relationship between the urban determinants of public health especially in low- and middle-income countries, which are currently facing similar challenges in terms of urbanization as well as growing levels of motorization and car use. As an example, in the past few years the number of registered cars in Latin American countries grew almost by 80 %, implying a 60 % rise in the motorization rate (cars per capita), approaching motorization levels in Asian countries (OICA, 2015). At the same time, this debate can also be informative cities in countries worldwide that are currently experiencing an increase in the prevalence of chronic health conditions such as obesity or diabetes tightly associated with the so-called adoption of a western lifestyle (Azeez, 2022).

## 3 Materials and methods

### 3.1 Data

This a cross-sectional study on data from the *Salud Urbana en América Latina* (SALURBAL) project (Quistberg et al., 2018). For the outcomes and individual-level covariates in this study we used harmonized data for adults 18 years or older in 178 cities from national health surveys. Health surveys collect data on health behaviors, risk factors, as well as other determinants or correlates of health such as socioeconomic status or living conditions. Data from the health surveys were gathered via national bureaus of statistics or other relevant government ministries. The included surveys varied by year depending on the country: Brazil (2013) (Instituto Brasileiro de Geografia e Estatística, 2013), Chile (2010) (Ministerio de Salud (MINSAL), 2010), Colombia (2007) (Ministerio de la Protección Social, 2007), Mexico (2012) (Instituto Nacionalde Salud Pública, 2012), and Peru (2016) (Instituto Nacional de Esta-dística e Informática, 2016). Additional details regarding the survey methods can be found in Supplemental Table S1. Cities in the SALURBAL project were defined as a single administrative unit (e.g., municipality) or a combination of adjacent administrative units (e.g., several municipalities) that are part of the urban extent determined from satellite imagery (Quistberg et al., 2019). Only individuals with complete information for all individual and city-level variables were included in this study. From the initial health survey sample of 199,260 individuals from 193 cities, we used 72,885 individuals for the obesity analysis and 87,448 individuals for the diabetes analysis residing in 178 cities (Supplemental Fig. S1). The significant reduction from the original survey sample is mostly explained by the fact that in some of the surveys the module relative either to weight status and or diabetes only included a sub-set of the sample. Characteristics of those included and excluded in each sample are presented in Supplemental Table S2.

### 3.2 Travel time assessment

The main exposure examined in this study is the city-level average travel time during the peak hour by car. Travel times were calculated in 2018 using the Google Maps Distance Matrix API (Google, 2018) by selecting 30 random origin-destination pairs in each city (considering only the urban extent instead of the entire administrative area) (Quistberg et al., 2019). Four morning peak hour measurements (6:30, 7:30, 8:30 and 9:30 am) and three peak hour afternoon measurements (5:30, 6:30, 7:30 pm) were taken for typical weekday (Tuesday-Thursday). For three cities (Santiago, Chile; Bogota, Colombia; and Sao Paulo, Brazil), estimated hourly travel time averages were compared to those from Uber Movement (Wu, 2018) for the same day of the week, hour, and year, suggesting high agreement (Pearson correlations = 0.869, 0.962, and 0.847, respectively).

### 3.3 Obesity and diabetes assessment

Obesity was defined as a Body Max Index (BMI) equal or over 30 kg/m^2^, calculated from measured height and weight. In sensitivity analyses we explored BMI as a continuous variable and BMI categories as defined by the World Health Organization (WHO) (n.d.): individuals with underweight (BMI <18.5 kg/m^2^), normal weight (BMI 18.5–24.9 kg/m^2^), overweight (BMI 25–29.99 kg/m^2^) and obesity (BMI ≥ 30 kg/m^2^). Diabetes was obtained by self-report of physician diagnosis.

### 3.4 Individual and city-level covariates

We included covariates at the individual and city levels that could be relevant in interpreting the associations between city-level travel time and individual obesity and diabetes. These covariates were identified based on previous studies (Sallis et al., 2012) and by constructing a directed acyclic graph (DAG) (Supplemental Fig. S2). DAGs are a theoretical models that aim to provide a visual overview of the causal research question and hypothesis and its context, making underlying relations explicit (Suttorp et al., 2015). The DAG was based on the authors' informed causal assumptions drawn by the evidence found in previous studies and summarized in the background section and was built using the online version of R-package ‘dagitty’.

At the individual level, we included the following self-reported variables extracted from the health surveys: sex (male/female), age (continuous), education level (less than primary, primary, secondary, university) and household car ownership (yes/no). At city-level, we included the following social and built environment variables compiled for each city as part of the SALURBAL project (Quistberg et al., 2019):

- Population density (persons per hectare): We divided the city population by the total built-up area in the city. Population was from 2016 and was extracted from national statistics agencies. Built-up area was extracted from 30 × 30m grid cells classified as urbanized in the Global Urban Footprint satellite imagery product (Esch et al., 2018).
- Intersection density (street intersections per square km): We divided the number of intersections by the total urban area of the city. Intersections were identified in Open Street Maps, and calculations were conducted using the OSMnx python library in 2018 (Boeing,2017).
- Adjusted gas price (% of monthly minimum wage): We measured the adjusted fuel price as the percentage of the country monthly minimum wage needed to purchase 10 gal of fuel. Gasoline prices in USD were obtained from country-specific sources and country minimum wages were obtained from the Salario Minimo platform in 2018 (Salario Minimo, n.d.).
- Presence of mass transit in the city (yes/no): We identified the presence of mass transit in the city considering Bus Rapid Transit (BRT) or subway. Data on BRT presence was extracted from the BRTData platform (Global BRT Data, n.d.) in 2017, and subway presence was complemented from city-specific official sources and OpenStreetMaps.
- Social Environment Index (SEI) (z-score): The SEI was calculated as a combination of country-specific census measures at city-level (Brazil 2010, Chile 2017, Colombia 2005, Mexico 2010 and Peru 2017). The index includes the proportion of households with piped water access inside the dwelling, the proportion of households connected to a public sewage network, the proportion of households with >3 people per room and the proportion of the population aged 25 or older who completed primary education or above. A higher score indicates a better social environment.

### 3.5 Statistical analyses

First, we described the bivariate associations between obesity and diabetes diagnosis with individual and city-level characteristics, by using Mann–Whitney *U* Test for continuous variables and Chi-square tests for categorical variables. Second, we used multilevel logistic regressions to examine the association between travel time and individual outcomes with a random intercept at city-level to account for clustering within cities. We built the main models incrementally: first, we estimated a model for each outcome only with the key exposure and adjusted by sex and age (Model 1); we then estimated a second model including the exposure while adjusting for other individual covariates such as education level and household car ownership (Model 2); third, we estimated a saturated model by adding the city-level covariates (Model 3). In all models we re-scaled the exposure (peak-hour travel time) to 10-minute units to facilitate interpretation. In the main models for obesity, normal weight is used as the reference category, excluding underweight, and we used obesity alone as the outcome to focus on the extreme category of BMI, thus excluding overweight. The saturated models were fitted based on the following:

$$\begin{array}{l} \text{logit}\;of\;P_{ij}=\beta 0+\beta 1*\;\text{City}\;-\;\text{level}\;\text{avg}\;.\text{travel}\;\text{timej}\;+\beta 2*\text{Sexij}\;+\beta 3*\text{Ageij}\;\\ \;+{\beta 4}^{*}\text{Edu}\text{.levelij}+\beta 5*\;\text{Car}\;\text{ownership}\text{.ij}\;+\beta 6*\text{Pop.densityj}\\ \;+{\beta 7}^{*}\text{Int}{\text{.densityj}\;+\beta 8}^{*}\text{Adj}{\text{.gas}\;\text{pricej}\;+\beta 9}^{*}\text{Presence}\;\text{of}\;\text{mass}\;\text{transitj}\;\\ \;+\beta 10*\text{SEIj}+\eta \text{j}+\text{eij} \end{array}$$

where *P_ij_* is the probability for each outcome in the *ith* individual, living in the *jth* city; *β* refer to fixed effects for the key exposure and all covariates, η_j_ refers to a random intercept at the city level, and e_ij_ is the residual error.

We also conducted several sensitivity analyses, presented in the Supplemental tables. First, we examined whether the association between the exposure and outcomes differed depending on city size. Thus, we stratified our analysis by city size (divided in categories based on built-up area tertiles). Second, we stratified the saturated models by sex and car ownership at the household level, given that the outcomes show significantly different proportions between males and females and between those who are part of households that own a car and those who are not. Third, given the diversity in health survey years from country to country, we also stratified models by country, and we also adjusted a model in which country is included as a fixed effect. Lastly, we explored if the association between the exposure and individual weight status varied if we used alternative operationalization of the outcome: we fitted a mixed effects linear regression with BMI as the outcome, a mixed effects logistic regression combining overweight and obesity as the outcome, and lastly, a mixed effects multinomial logistic regression with BMI categories as the outcome.

Data processing and analyses were conducted using ‘pandas’ and ‘TableOne’ Python libraries for the descriptive part and SPSS version 21 (IBM Corp. Released 2012. IBM SPSS Statistics for Windows, Version 21.0. Armonk, NY, USA) for the models.

## 4 Results

We analyzed data from a total of 72,885 survey respondents for obesity and 87,448 survey respondents for diabetes from a total of 178 cities. Individuals included in the obesity sample were more frequently females (58 %) and had a median age of 39 years (Table 1). Secondary education was the most common education level attained among respondents (37.5 %), followed by primary (30.1 %), less than primary (20.2 %) and university (12.2 %). Most individuals in the obesity sample were part of households that did not own a car (67.2 %). Individuals in the obesity sample lived in cities with a median peak-hour travel time of 23.3 min, a median population density of 84.4 persons per hectare, a median of 87.7 intersections per square km and an adjusted gas price of 3.56 % of the monthly minimum wage to buy 10 gal of vehicle fuel. Slightly less than half of the individuals in this sample lived in cities were mass transit was present (48.9 %). Lastly, the median SEI (z-score) for cities in the sample was 0.12. With minor exceptions, the characteristics of the diabetes sample were similar to the obesity sample (Table 2). Supplemental Table S3 presents obesity and diabetes diagnosis by individual and city characteristics.

Overall, 25 % of the obesity sample correspond to individuals with obesity (*n* = 18,155) (Supplemental Table S3). Individuals with obesity were more likely to be female, older, and with lower educational attainment than those without obesity (Table 1). Individuals with obesity tended to live in cities with slightly shorter average peak-hour travel times, lower population density and slightly higher intersection density. The percent of the monthly minimum wage needed to buy 10 gal of gasoline was higher and mass transit was less common in cities where individuals with obesity lived.

Overall, 6.4 % of the sample had a diabetes diagnosis (*n* = 5626) (Supplemental Table S3). Individuals diagnosed with diabetes were more likely to be female, older, and with lower educational attainment than those without diabetes (Table 2). Individuals with diabetes lived in cities with slightly longer average travel times, and with lower population and intersection densities. The percent of the monthly minimum wage needed to buy 10 gal of gasoline and the Social Environment Index was higher in cities where individuals with diabetes lived.

Table 3 shows results of the association between peak-hour travel time and individual obesity and diabetes. When adjusting only for age and sex (Model 1), the association between city-level travel time and obesity was negative (OR: 0.907) but the confidence intervals were imprecise (0.821, 1.001). When adjusting by individual education level and household car ownership (Model 2), the direction of the association remained negative (OR: 0.903) and the confidence intervals increased precision (0.821, 0.993). Lastly, when adjusting for city-level covariates in the saturated model (Model 3), the association between city-level peak-hour travel time and individual obesity changed to positive, but the confidence intervals widened (OR: 1.006 [0.920, 1.099]). Among the individual and city-level covariates, we observed in the saturated model that age, primary education, car ownership, city intersection density and adjusted gas price, showed a significant positive association with individual-level obesity. Conversely, sex (male), university education, city-level population density, and the social environment index showed a negative association with individual obesity.

In the case of diabetes, when adjusting only by sex and age (Model 1), the association between the city-level travel time and diabetes was negative (OR: 0.995) and showed wide confidence intervals (0.925, 1.071). When adjusting for education level and household car ownership (Model 2), we observed a positive but low and uncertain association between city-level peak-hour travel time and diabetes (OR: 1.006 [0.937, 1.076]). Finally, when adjusting for city-level covariates in the saturated model, the association between the key exposure and diabetes was negative and presented wide confidence intervals (OR: 0.991 [0.933, 1.052]). Some of the associations found between the individual and city-level covariates and diabetes were similar to those observed in the obesity model, while others changed. Sex (male), higher education level and population density continued to show a negative association with diabetes. Age and city-level adjusted gas price continued to show a positive association with this outcome. However, primary education now showed a negative association with the outcome, the confidence intervals of the association between car ownership and diabetes widened resulting in an uncertain positive association, city-level intersection density now showed a negative association with individual-level diabetes, and the social environment index showed a positive association with diabetes.

Graphs describing the projected probabilities for each outcome relative to the key exposure contrasted by population density are presented in Supplementary Figs. S3 and S4. Similar results in terms of magnitude and confidence were observed in the different sensitivity analyses, which include the saturated models stratified by city size, sex, household car ownership, country (Supplemental Tables S4-S8), and different outcome categorizations for weight status (Supplemental Table S9).

## 5 Discussion

Latin American cities are currently faced with an unprecedented increase in vehicle motorization rates, higher private car use and long daily travel times. At the same time, obesity and diabetes are considered two of the main public health challenges in the region, partially related to higher levels of sedentarism. In this paper we aimed to examine the association between city-level travel time during peak hour and residents’ individual-level odds of obesity and diabetes. For this purpose, we used a large sample of individuals residing in 178 cities over 100,000 inhabitants in five Latin American countries. Contrary to our initial hypotheses, we did not observe an association between city-level peak-hour travel time and either individual obesity or diabetes in the examined cities, while we observed interesting associations with some of the covariates included in the study.

Despite previous evidence of the associations between individual travel time and individual odds of obesity (López-Zetina et al., 2006) and other metabolic-related health issues (Sugiyama et al., 2016), our results suggest that the relationship between travel time at city-level and individual obesity and diabetes may be more complex in the selected Latin American cities. Individual's time spent in vehicles was a hypothesized pathway between city-level travel times and the outcomes in this study. One possible explanation for the unexpected results is that Latin American cities have generally remained dense and compact (Inostroza et al., 2013) and with high street density. In turn, walking and public transportation as modes of transport have remained relatively high (Banco de Desarrollo de América Latina, 2017; Delclòs-Alió et al.,2021) and in many cities car restrictions could lead to multimodal transportation and active travel (Rivas et al., 2019), thereby ensuring relevant levels physical activity through walking for transport (de Sáet al., 2017; Ferrari et al., 2020; Lemoine et al., 2016) independently from long travel times at the city level. In addition, car ownership and use is strongly associated with higher SES in Latin America (Ferrari et al., 2020; Gandelman et al., 2019), and at the same time individuals in higher SES groups are currently more likely to have healthier diets and engage in more leisure physical activity (Gémez et al., 2019; Werneck et al., 2019), which could be compensating for sedentary time spent in cars. Along this line, future studies could take a step back in the hypothesized causal pathway and look specifically at the relationship between city-level travel times and dietary patterns on city residents in low- and middle-income contexts. As an example, a recent study in the Latin American context suggested a causal relationship between long travel times and the consumption of ultra-processed food, through a lower time availability to prepare food at home (Langellier et al., 2019).

We had also hypothesized that the association between city-level travel time and individual odds of obesity and diabetes could be related not only to time spent in motor vehicles, but also to being exposed to the diverse externalities of longer city-level travel times. This indirect pathway would consider the effects of having cars circulating for longer periods of time (pollution, noise, perception of safety). This could result in increased emissions, noise or personal safety concerns and could also discourage daily physical activity levels among urban residents or even affect them directly (Jerrett et al., 2014; Malambo et al., 2018; Oftedal et al., 2015; Pyko et al., 2015). However, although our research design did not actually measure traffic-related repercussions such as pollution, noise or personal safety, the lack of an association between city-level travel time and the individual-level outcomes the 178 cities analyzed in this study would not support this indirect pathway, in line with other studies (Fioravanti et al., 2018). A possible explanation is that cities in Latin America are experiencing unprecedented urbanization rates that could be resulting in built environments that are different from other world regions (Inostroza et al., 2013), which in turn may have different implications in terms of the relationship between individuals and their surrounding environment In this sense, there may be other relevant characteristics of Latin American cities that we did not consider, which future research could explore further. An example would be the role of spatial inequality as a potential modifier of the exposure of individuals to traffic and its environmental and health-related repercussions. Latin American cities are among the most unequal in the world, which has important health repercussions (Bilal et al., 2019), and thus it is possible that not all city residents are affected equally by the externalities of a prolonged presence of cars at city-level. In this line, future studies could incorporate heterogeneity in the exposure to the negative repercussions of the presence of cars in Latin American cities.

We did, however, find relevant associations between city-level covariates and individual-level obesity and diabetes that could be informative to future studies. For example, we found a consistent negative association between population density and both individual odds of obesity and diabetes, which could be in line with previous studies that have focused on the key role that density plays as a component of walkability. More specifically, population density is traditionally linked to built-up density as well as density of destinations, which results in shorter distances between day-to-day origins and destinations (Ewing and Cervero, 2010). In turn, this leads to a lower dependence on motorized transport and, consequently, to higher use of physically active modes of transport such as walking or cycling, which at the same time are associated with lower odds of overweight and diabetes (Glazier et al., 2014; Slater et al., 2013; Zhao and Kaestner, 2010). We also observed a positive association between adjusted gas price and individual odds of both obesity and diabetes. A plausible explanation for this result could be the fact that larger cities tend to present higher gas prices, while at the same time previous studies have linked the transition from rural to urban lifestyles in Latin America with the adoption of more sedentary behavior (Cuevas et al., 2009).

In addition, we also observed some unexpected contradictory associations at the city-level: for example, intersection density showed a positive association with individual-level obesity, which was contrary to what was found in other settings (Nichani et al., 2020), and a negative association with diabetes. These conflicting results could be explained by the fact that in some areas, high intersection densities without considering other qualities of urban form could be related not with higher levels of walkability but with higher levels of traffic congestion, which could also have a negative effect on health-related issues. For example, a recent study focusing on a large sample of residents in 230 Latin American cities it was found that higher sub-city intersection density and the presence of mass transit were both associated with higher odds of having hypertension (Avila-Palencia et al., 2022). In that study, it was suggested that intersection density could be associated with traffic, and in turn with air pollution, heat, and noise, which could be factors related to cardiovascular risk. On the contrary, the level of city socioeconomic development showed a negative association with individual odds of obesity and a positive association with diabetes. These associations are particularly challenging to interpret, considering the undergoing nutritional transition in the region. While a higher socioeconomic level may had been initially associated with nonhealthy diet, over time the situation may have been inverted (Cuevas et al., 2009; Jaacks et al., 2019). Consequently, additional studies are required to disentangle how the built and social environment at the city level may be impacting obesity and diabetes differently in the Latin American context.

This study has several strengths. First, we used individual survey data from five Latin American countries and >170 cities with over 100,000 inhabitants, resulting in two large samples consisting of 72,885 and 87,448 individuals. To the best of our knowledge, this is the largest study evaluating the association of city-level travel time and individual odds of obesity and diabetes in the Latin American context. Second, our key exposure was based on an innovative calculation based on publicly available data extracted from Google Maps Distance Matrix API (Google, 2018), which showed high agreement with other travel time estimates for three cities in Latin America, has been also used in previous studies (F. Wang and Xu, 2011; Wu, 2018), and can be easily replicated elsewhere. Third, we estimated mixed-effects models to be able to account for the multilevel nature of the hypothesized association between a key exposure at the city level and individual outcomes (Diez-Roux, 2000), while at the same time including individual and city-level covariates and including a random effect at the city-level.

This study also has several limitations to take into consideration. First, we used a cross-sectional design, and therefore no causality can be interpreted from the associations found in the results. Second, the outcomes explored in the analysis preceded the key exposure. While data on average travel times form Google Maps was calculated in 2018, survey years providing data on obesity and diabetes ranged from 2007 to 2016. Also, health surveys used to evaluate the outcomes and individual covariates varied in years from country to country, and some of them could present specific differences in data collection. However, these two limitations are not likely to have a significant impact in our results, considering that sensitivity analyses controlling by country (and survey year) did not show any significant differences in the association between the exposure and the outcomes. Fourth, while our hypothesis was based on the notion that individual odds of obesity or diabetes could be also affected by living in a city with longer travel times, we could not assess possible spatial heterogeneity of exposures across individuals in the same city. This could trigger future studies that include residential location and characteristics in the examination of the association between car use at the city level and individual odds of obesity and diabetes. Fifth, the ascertainment of the outcomes, especially diabetes, could be affected by the fact that in low and middle-income countries certain diagnoses may be less reliable than in high-income countries (Lerner et al., 2013; Rubinstein et al., 2014). Sixth, we could not adjust for individual travel behavior patterns, which are likely to affect the association between travel time and obesity and diabetes. This is especially important in terms of the distinction between private vehicle users and public transit users, although we included car ownership at the household level both as an adjustment variable and we stratified the sample by car ownership as a sensitivity analysis, and no changes were observed in the associations. Finally, since this is an observational study, residual confounding could still be present despite our effort to select key variables as potential confounders in our models.

In conclusion, we did not find any association between city-level travel time during the peak hour and individual odds of obesity or diabetes among adults in our study. We did, however, find interesting associations between certain features of the urban form and the two outcomes in our study, which suggests that policies aiming at reducing the prevalence of such chronic conditions in Latin America and in other low- and middle-income settings need to complement health-related interventions with policies and strategies at promoting urban design that are conducive of physical activity. The fact that we did not find any evidence of city-level travel time and the development of obesity or diabetes of city dwellers in Latin America should encourage more studies that aim to disentangle the complex relationship between the prolonged presence of cars in cities and individual obesity and diabetes, considering both the continued increase of car ownership and use as well as the prevalence of obesity and diabetes in developing countries worldwide.

## Supplementary Material

Supplementary Data

## Figures and Tables

**Table 1:** Characteristics of the obesity sample.

	Total obesity sample (*n* = 72,885)	Obesity		p-Value ^a^
		No (*n* = 54,730)	Yes (*n* = 18,155)	
Individual characteristics				
Sex				<0.001
Female	42,276 (58.0)	30,568 (55.9)	11,708 (64.5)	
Male	30,609 (42.0)	24,162 (44.2)	6447 (35.5)	
Age	39 [29,53]	38 [28,52]	43 [34,55]	<0.001
Education				<0.001
< Primary	14,684 (20.2)	10,459 (19.1)	4225 (23.3)	
Primary	21,963 (30.1)	15,879 (29.0)	6084 (33.5)	
Secondary	27,345 (37.5)	21,402 (39.1)	5943 (32.7)	
University	8893 (12.2)	6990 (12.8)	1903 (10.5)	
Household car ownership				0.249
No	48,937 (67.2)	36,811 (67.3)	12,126 (66.8)	
Yes	23,948 (32.9)	17,919 (32.7)	6029 (33.2)	
City characteristics				
Peak-hour travel time (min.)	23.3 [16.7,33.3]	24.7 [16.8,33.8]	22.21 [15.5,33.3]	<0.001
Population density (pop./ha)	84.4 [63.1, 113.4]	84.7 [64.8, 113.4]	80.0 [61.9106.2]	<0.001
Intersection density (n/km^2^)	87.7 [77.4111.7]	87.7 [77.4111.7]	88.0 [77.5112.3]	<0.001
Adjusted gas price (% of monthly min. wage)	3.56 [3.36,3.97]	3.56 [3.36,3.97]	3.62 [3.36,6.66]	<0.001
Presence of mass transit				<0.001
No	37,270 (51.1)	27,267 (49.8)	10,003 (55.1)	
Yes	35,615 (48.9)	27,463 (50.2)	8152 (44.9)	
Social				
Environment Index (z-score)	0.12 [−0.32,0.46]	0.12 [−0.32,0.46]	0.06 [−0.32,0.46]	0.841

Values are show as n (%) or p50 [p25, p75].

a Chi-square test for categorical variables, Mann-Whitney U Test for continuous variables.

**Table 2:** Characteristics of the diabetes sample.

	Total diabetes sample (n = 87,448)	Diabetes		p-Value^a^
		No (*n* = 81,822)	Yes (*n* = 5626)	
Individual characteristics				
Sex				<0.001
Female	51,176 (58.5)	47,684 (58.3)	3492 (62.1)	
Male	36,272 (41.5)	34,138 (41.7)	2134 (37.9)	
Age	40 [30,53]	39 [29,51]	59 [49,68]	<0.001
Education				
<Primary	17,438 (19.9)	15,124 (18.5)	2314 (41.1)	<0.001
Primary	26,736 (30.6)	25,056 (30.6)	1680 (29.9)	
Secondary	32,474 (37.1)	31,322 (38.3)	1152 (20.5)	
University	10,800 (12.4)	10,320 (12.6)	480 (8.5)	
Household car ownership				
No	60,972 (69.7)	57,026 (69.7)	3946 (70.1)	0.493
Yes	26,476 (30.3)	24,796 (30.3)	1680 (29.9)	
City characteristics				
Peak-hour travel time (min.)	22.2 [15.0,32.5]	22.2 [15.0,32.5]	22.3 [15.8,33.3]	0.002
Population density (pop./ha)	91.8 [66.3, 141.6]	91.8 [67.2, 142.9]	80.0 [61.9, 113.4]	<0.001
Intersection density (n/km^2^)	98.7 [79.3122.7]	100.0 [79.3122.7]	88.0 [77.5111.7]	<0.001
Adjusted gas price (% of monthly min. wage)	3.54 [2.74,3.97]	3.54 [2.70,3.97]	3.62 [3.28,6.66]	<0.001
Presence of mass transit				
No	44,947 (51.4)	42,070 (51.4)	2877 (51.1)	0.696
Yes	42,501 (48.6)	39,752 (48.6)	2749 (48.9)	
Social				
Environment Index (z-score)	0.19 [−0.24,0.53]	0.16 [−0.25,0.52]	0.28 [−0.19,0.53]	<0.001

Values are show as n (%) or p50 [p25, p75].

a Chi-square test for categorical variables, Mann–Whitney *U* Test for continuous variables.

**Table 3:** Odds ratios of obesity and diabetes associated with city-level peak hour travel time and individual and city co-variates.

	Model 1	Model 2	Model 3
	OR (95 % CI)	OR (95 % CI)	OR (95 % CI)
Obesity (*n* = 44,041)^a^			
Peak-hour travel time (10 min units)	0.907 (0.821, 1.001)	0.903 (0.821, 0.993)*	1.006 (0.920, 1.099)
Male (Ref. = Female)	0.717 (0.688, 0.747)*	0.707 (0.678, 0.736)*	0.707 (0.678, 0.736)*
Age	1.025 (1.024, 1.026)*	1.026 (1.024, 1.027)*	1.026 (1.024, 1.027)*
University (Ref. = Less than primary)		0.812 (0.750, 0.879)*	0.820 (0.758, 0.888)*
Secondary (Ref. = Less than primary)		0.984 (0.924, 1.048)	0.999 (0.938, 1.065)
Primary (Ref. = Less than primary)		1.249 (1.174, 1.329)*	1.249 (1.174, 1.329)*
Car ownership (Yes)		1.257 (1.199, 1.319)*	1.244 (1.186, 1.304)*
Population density (pop./ha)			0.995 (0.993, 0.996)*
Intersection density (n/sq. km)			1.006 (1.003, 1.009)*
Adjusted gas price (% of monthly min. wage)			1.158 (1.111, 1.207)*
Presence of mass transit (Yes)			0.926 (0.756, 1.134)
Social Environment Index (z- score)			0.898 (0.811, 0.994)*
Diabetes (*n* = 87,448)			
Peak-hour travel time (10 min units)	0.995 (0.925, 1.071)	1.004 (0.937, 1.076)	0.991 (0.933, 1.052)
Male (Ref. = Female)	0.863 (0.814, 0.915)*	0.872 (0.822, 0.925)*	0.872 (0.823, 0.925)*
Age	1.060 (1.058, 1.062)*	1.057 (1.055, 1.059)*	1.057 (1.055, 1.059)*
University (Ref. = Less than primary)		0.639 (0.571, 0.715)*	0.643 (0.575, 0.720)*
Secondary (Ref. = Less than primary)		0.718 (0.660, 0.782)*	0.730 (0.670, 0.795)
Primary (Ref. = Less than primary)		0.928 (0.861, 1.000)*	0.924 (0.858, 0.996)*
Car ownership (Yes)		1.062 (0.992, 1.137)	1.042 (0.973, 1.115)
Population density (pop./ha)			0.999 (0.997, 1.000)*
Intersection density (int/sq. km)			0.997 (0.995, 0.999)*
Adjusted gas price (% of monthly min. wage)			1.124 (1.089, 1.161)*
Presence of mass transit (Yes)			1.019 (0.884, 1.173)
Social Environment Index (z-score)			1.103 (1.019, 1.194)*

Mixed effects logistic regressions with random effects at the city level. Outcomes: obesity and diabetes. Key exposure: city-level travel time during peak hour in 10-min units. Model 1: adjusted by age and sex. Model 2: adjusted by age, sex, education level, household car ownership. Model 3: adjusted by age, sex, education level, household car ownership, city population density, intersection density, adjusted gas price, presence of mass transit and social environment index.

a Normal weight used as reference category (BMI between 18.5 and 24.9 kg/m^2^); underweight and overweight excluded.

* *p*-value <0.05.

## References

[R1] Akbar PA, Duranton G (2017). Measuring the cost of congestion in highly congested city: Bogota.

[R2] Alderete TL, Chen Z, Toledo-Corral CM, Contreras ZA, Kim JS, Habre R, Chatzi L, Bastain T, Breton CV, Gilliland FD (2018). Ambient and traffic-related air pollution exposures as novel risk factors for metabolic dysfunction and type 2 diabetes. Current Epidemiology Reports 5.

[R3] Anderson ML, Lu F, Yang J (2019). Physical activity and weight following car ownership in Beijing, China: Quasi-experimental cross sectional study. The BMJ.

[R4] Avila-Palencia I, Miranda JJ, Rodríguez DA, Moore K, Gouveia N, Moran MR, TeixeiraCaiaffa W, DiezRoux AV (2022). Associations of urban environment features with hypertension and blood pressure across 230 Latin American cities. Environmental Health Perspectives.

[R5] Azeez TA (2022). Obesity in Africa: The challenges of a rising epidemic in the midst of dwindling resources. Obesity Medicine.

[R6] Banco de Desarrollo de America Latina (2017). Crecimiento urbano y acceso a oportunidades: un desafío para America Latina.

[R7] Bilal U, Alazraqui M, Caiaffa WT, Lopez-Olmedo N, Martinez-Folgar K, Miranda JJ, Rodriguez DA, Vives A, Diez-Roux AV (2019). Inequalities in life expectancy in six large Latin American cities from the SALURBAL study: An ecological analysis. The Lancet Planetary Health.

[R8] Boeing G (2017). OSMnx: New methods for acquiring, constructing, analyzing, and visualizing complex street networks. Computers, Environment and Urban Systems.

[R9] Carrillo-Larco RM, Bernabe-Ortiz A, Pillay TD, Gilman RH, Sanchez JF, Poterico JA, Quispe R, Smeeth L, Miranda JJ (2016). Obesity risk in rural, urban and rural-to-urban migrants: Prospective results of the PERU MIGRANT study. International Journal of Obesity.

[R10] Celis-Morales CA, Lyall DM, Petermann F, Anderson J, Ward J, Iliodromiti S, Mackay DF, Welsh P, Bailey MES, Pell J, Sattar N (2019). Do physical activity, commuting mode, cardiorespiratory fitness and sedentary behaviours modify the genetic predisposition to higher BMI? Findings from a UK biobank study. International Journal of Obesity.

[R11] Chapman L (2007). Transport and climate change: A review. Journal of Transport Geography.

[R12] Christian TJ (2012). Trade-offs between commuting time and health-related activities. Journal of Urban Health.

[R13] Clark C, Sbihi H, Tamburic L, Brauer M, Frank LD, Davies HW (2017). Association of long-term exposure to transportation noise and traffic-related air pollution with the incidence of diabetes: A prospective cohort study. Environmental Health Perspectives.

[R14] Cuevas A, Alvarez V, Olivos C (2009). The emerging obesity problem in Latin America. Expert Review of Cardiovascular Therapy.

[R15] Delclòs-Alió X, Rodríguez DA, Medina C, Miranda JJ, Ávila-Palencia I, Targa F, Moran MR, Sarmiento OL, Quistberg DA (2021). Walking for transportation in large Latin American cities: Walking-only trips and total walking events and their sociodemographic correlates. Transport Reviews.

[R16] Dendup T, Feng X, Clingan S, Astell-Burt T (2018). Environmental risk factors for developing type 2 diabetes mellitus: A systematic review. International Journal of Environmental Research and Public Health.

[R17] Diez-Roux AV (2000). Multilevel analysis in public health research. Annual Review of Public Health.

[R18] Divney AA, Murillo R, Rodriguez F, Mirzayi CA, Tsui EK, Echeverria SE (2019). Diabetes prevalence by leisure-,transportation-,and occupation-based physical activity among racially/ethnically diverse U.S. adults. Diabetes Care.

[R19] Esch T, Bachofer F, Heldens W, Hirner A, Marconcini M, Palacios-Lopez D, Roth A, Zeidler J, Dech S, Gorelick N, Üreyen S (2018). Where we live-A summary of the achievements and planned evolution of the global urban footprint. Remote Sensing.

[R20] Euler R, Jimenez EY, Sanders S, Kuhlemeier A, Van Horn ML, Cohen D, Gonzales-Pacheco D, Kong AS (2019). Rural-urban differences in baseline dietary intake and physical activity levels of adolescents. Preventing Chronic Disease.

[R21] Ewing R, Cervero R (2010). Travel and the built environment: A meta-analysis. Journal of the American Planning Association.

[R22] Ewing R, Schmid T, Killingsworth R, Zlot A, Raudenbush S (2008). Relationship between urban sprawl and physical activity, obesity, and morbidity. American Journal of Health Promotion.

[R23] Ewing R, Meakins G, Hamidi S, Nelson AC (2014). Relationship between urban sprawl and physical activity, obesity, and morbidity - Update and refinement. Health and Place.

[R24] Ferrari GLdM, Kovalskys I, Fisberg M, GómezSalas G, Rigotti A, Cortes Sanabria LY, Alberico C (2020). Socio-demographic patterns of public, private and active travel in Latin America: Cross-sectional findings from the ELANS study. Journal of Transport and Health.

[R25] Fioravanti S, Cesaroni G, Badaloni C, Michelozzi P, Forastiere F, Porta D (2018). Traffic-related air pollution and childhood obesity in an Italian birth cohort. Environmental Research.

[R26] Frank LD, Andresen MA, Schmid TL (2004). Obesity relationships with community design, physical activity, and time spent in cars. American Journal of Preventive Medicine.

[R27] Frederick C, Riggs W, Gilderbloom JH (2018). Commute mode diversity and public health: A multivariate analysis of 148 US cities.

[R28] Gandelman N, Serebrisky T, Suóarez-Alemaón A (2019). Household spending on transport in Latin America and the Caribbean: A dimension of transport affordability in the region. Journal of Transport Geography.

[R29] Garrido-Méndez A, Díaz X, Martinez MA, Leiva AM, Álvarez C, Campillo RR, Cristi-Montero C, Rodríguez F, Salas Bravo C, Durán E, Labraña AM (2017). Mayores niveles de transporte activo se asocian a un menor nivel de adiposidad y menor riesgo de obesidad: Resultados de la encuesta nacional de salud 2009-2010. Revista Medica de Chile.

[R30] Gémez G, Fisberg RM, Previdelli ÁN, Sales CH, Kovalskys I, Fisberg M, Herrera-Cuenca M, Sanabria LYC, García MCY, Torres RGP, Rigotti A (2019). Diet quality and diet diversity in eight Latin American countries: Results from the Latin American study of nutrition and health (ELANS). Nutrients.

[R31] Glazier RH, Creatore MI, Weyman JT, Fazli G, Matheson FI, Gozdyra P, Moineddin R, Shriqui VK, Booth GL (2014). Density, destinations or both? A comparison of measures of walkability in relation to transportation behaviors, obesity and diabetes in Toronto, Canada. PLoS ONE.

[R32] Global BRT Data, n.d.Global BRT Data. (n.d.) https://brtdata.org/.

[R33] Google (2018). Distance Matrix API.

[R34] Gössling S, Choi A, Dekker K, Metzler D (2019). The social cost of automobility, cycling and walking in the European Union. Ecological Economics.

[R35] Heisler M, Kaselitz E, Rana GK, Piette JD (2016). Diabetes prevention interventions in Latin American countries: A scoping review. Current Diabetes Reports.

[R36] Hoehner CM, Barlow CE, Allen P, Schootman M (2012). Commuting distance, cardiorespiratory fitness, and metabolic risk. American Journal of Preventive Medicine.

[R37] Inostroza L, Baur R, Csaplovics E (2013). Urban sprawl and fragmentation in Latin America: A dynamic quantification and characterization of spatial patterns. Journal of Environmental Management.

[R38] INRIX (2019). INRIX 2018 global traffic scorecard.

[R39] Instituto Brasileiro de Geografia e Estatística (2013). Pesquisa Nacional de Saúde 2013:percepçao do estado de saúde, estilos de vida e doenças cronicas - Brasil, grandes regioes eunidades da federação.

[R40] Instituto Nacional de Estadística e Informatica (2016). Encuesta demografica y de SaludFamiliar ENDES 2016 de Peru.

[R41] Instituto Nacional de Salud Pública (2012). Encuesta Nacional de Salud y Nutricion(ENSANUT) 2012 de Móxico.

[R42] Jaacks LM, Vandevijvere S, Pan A, Mc Gowan CJ, Wallace C, Imamura F, Mozaffarian D, Swinburn B, Ezzati M (2019). The obesity transition: Stages of the global epidemic. The Lancet Diabetes and Endocrinology.

[R43] Jerrett M, Mc Connell R, Wolch J, Chang R, Lam C, Dunton G, Gilliland F, Lurmann F, Islam T, Berhane K (2014). Traffic-related air pollution and obesity formation in children: A longitudinal, multilevel analysis. Environmental Health: A Global Access Science Source.

[R44] Jerrett M, Brook R, White LF, Burnett RT, Yu J, Su J, Seto E, Marshall J, Palmer JR, Rosenberg L, Coogan PF (2017). Ambient ozone and incident diabetes: A prospective analysis in a large cohort of african american women. Environment International.

[R45] Kellstedt DK, Washburn DJ, Lee S, Gwarzo I, Ahenda P, Maddock JE (2021). Household motor vehicle ownership and obesity among indian females and males: 2005-2016. International Health.

[R46] Langellier BA, Kuhlberg JA, Ballard EA, Slesinski SC, Stankov I, Gouveia N, Meisel JD, Kroker-Lobos MF, Sarmiento OL, Caiaffa WT, Diez Roux AV (2019). Using community-based system dynamics modeling to understand the complex systems that influence health in cities: The SALURBAL study. Health and Place.

[R47] Lemoine PD, Sarmiento OL, Pinzoón JD, Meisel JD, Montes F, Hidalgo D, Pratt M, Zambrano JM, Cordovez JM, Zarama R (2016). TransMilenio, a scalable bus rapid transit system for promoting physical activity. Journal of Urban Health.

[R48] Lerner AG, Bernabe-Ortiz A, Gilman RH, Smeeth L, Miranda JJ (2013). The ‘rule of halves’does not apply in Peru: Awareness, treatment, and control of hypertension and diabetes in rural, urban and rural-to-urban migrants. Critical Pathways in Cardiology.

[R49] Lopez-Zetina J, Lee H, Friis R (2006). The link between obesity and the built environment. Evidence from an ecological analysis of obesity and vehicle miles of travel in California. Health and Place.

[R50] Malambo P, De Villiers A, Lambert EV, Puoane T, Kengne AP (2018). Associations of perceived neighbourhood safety from traffic and crime with overweight/obesity among South African adults of lowsocioeconomic status.

[R51] Mc Cormack GR, Virk JS (2014). Driving towards obesity: A systematized literature review on the association between motor vehicle travel time and distance and weight status in adults. Preventive Medicine.

[R52] Salario Minimo, n.d.Salario Minimo. (n.d.) Salario Mínimo en Latinoamerica ¿en donde se gana maós.

[R53] Ministerio de la Protección Social (2007). Encuesta Nacional de Salud de Colombia.

[R54] Ministerio de Salud (MINSAL) (2010). Encuesta Nacional de Salud de Chile 2009-2010.

[R55] Mueller N, Rojas-rueda D, Basagana X, Cirach M, Cole-hunter T, Dadvand P, Donaire-gonzalez D, Foraster M, Gascon M, Martinez D, Tonne C (2017). Urban and transport planning related exposures and mortality: A health impact assessment for cities. Environmental Health Perspectives.

[R56] NCD Risk Factor Collaboration (NCD-RisC) - Americas Working Group (2020). Trends in cardiometabolic risk factors in the Americas between 1980 and 2014: A pooled analysis of population-based surveys. The Lancet Global Health.

[R57] Nichani V, Turley L, Vena JE, McCormack GR (2020). Associations between the neighbourhood characteristics and body mass index, waist circumference, and waist-to-hip ratio: Findings from Alberta’s Tomorrow Project. Health and Place.

[R58] Oftedal B, Krog NH, Pyko A, Eriksson C, Graff-Iversen S, Haugen M, Schwarze PE, Pershagen G, Aasvang GM (2015). Road traffic noise and markers of obesity - A population-based study. Environmental Research.

[R59] OICA (2015). World Vehicles in Use 2005-2015.

[R60] Parra DC, Lobelo F, Goómez LF, Rutt C, Schmid T, Brownson RC, Pratt M (2009). Household motor vehicle use and weight status among colombian adults: Are we driving our way towards obesity?. Preventive Medicine.

[R61] Patil GR, Sharma G (2021). Overweight/obesity relationship with travel patterns, socioeconomic characteristics, and built environment. Journal of Transport and Health.

[R62] Pereira MF, Almendra R, Vale DS, Santana P (2020). The relationship between built environment and health in the Lisbon Metropolitan area – Can walkability explain diabetes’ hospital admissions?. Journal of Transport and Health.

[R63] Popkin BM, Reardon T (2018). Obesity and the food system transformation in Latin America. Obesity Reviews.

[R64] Pyko A, Eriksson C, Oftedal B, Hilding A, Ostenson CG, Krog NH, Julin B, Aasvang GM, Pershagen G (2015). Exposure to traffic noise and markers of obesity. Occupational and Environmental Medicine.

[R65] Quistberg DA, Roux AVD, Bilal U, Moore K, Ortigoza A, Rodríguez DA, Sarmiento OL, Frenz P, Friche AA, Caiaffa WT, Vives A (2018). Building a data platform for cross-country urban health studies: The SALURBAL study. Journal of Urban Health.

[R66] Quistberg DA, Diez Roux AV, Bilal U, Moore K, Ortigoza A, Rodriguez DA, Sarmiento OL, Frenz P, Friche AA, Caiaffa WT, Vives A (2019). Building a data platform for cross-country urban health studies: The SALURBAL study. Journal of Urban Health.

[R67] Renzi M, Cerza F, Gariazzo C, Agabiti N, Cascini S, Di Domenicantonio R, Davoli M, Forastiere F, Cesaroni G (2018). Air pollution and occurrence of type 2 diabetes in a large cohort study. Environment International.

[R68] Rivas ME, Suárez-Alemán A, Serebrisky T (2019). Urban transport policies in Latin America and the Caribbean. Where we are, how we got here, what lies ahead.

[R69] Rodrigue J-P, Comtois C, Slack B (2013). The geography of transport systems.

[R70] Roswall N, Raaschou-Nielsen O, Jensen SS, Tjønneland A, Sørensen M (2018). Long-term exposure to residential railway and road traffic noise and risk for diabetes in a Danish cohort. Environmental Research.

[R71] Rubinstein A, Gutierrez L, Beratarrechea A, Irazola VE (2014). Increased prevalence of diabetes in Argentina is due to easier health care access rather than to an actual increase in prevalence. PLoS ONE.

[R72] de Sá TH, de Rezende LFM, Borges MC, Nakamura PM, Anapolsky S, Parra D, Adami F, Monteiro CA (2017). Prevalence of active transportation among adults in Latin America and the Caribbean: A systematic review of population-based studies. Revista Panamericana de Salud Pública.

[R73] Sallis JF, Floyd MF, Rodríguez DA, Saelens BE (2012). The role of built environments in physical activity, obesity, and CVD. Circulation.

[R74] Shoup D (2015). Putting a cap on parking requirements.

[R75] Slater SJ, Nicholson L, Chriqui J, Barker DC, Chaloupka FJ, Johnston LD (2013). Walkable communities and adolescent weight. American Journal of Preventive Medicine.

[R76] Steell L, Garrido-Mendez A, Petermann F, Díaz-Martínez X, Martinez MA, Leiva AM, Salas-Bravo C, Alvarez C, Ramirez-Campillo R, Cristi-Montero C, Rodríguez F (2018). Active commuting is associated with a lower risk of obesity, diabetes and metabolic syndrome in Chilean adults. Journal of Public Health (United Kingdom).

[R77] Sugiyama T, Ding D, Owen N (2013). Commuting by car: Weight gain among physically active adults. American Journal of Preventive Medicine.

[R78] Sugiyama T, Wijndaele K, Koohsari MJ, Tanamas SK, Dunstan DW, Owen N (2016). Adverse associations of car time with markers of cardio-metabolic risk. Preventive Medicine.

[R79] Sugiyama T, Chandrabose M, Homer AR, Sugiyama M, Dunstan DW, Owen N (2020). Car use and cardiovascular disease risk: Systematic review and implications for transport research. Journal of Transport and Health.

[R80] Suttorp MM, Siegerink B, Jager KJ, Zoccali C, Dekker FW (2015). Graphical presentation of confounding in directed acyclic graphs. Nephrology Dialysis Transplantation.

[R81] Wang F, Xu Y (2011). Estimating O-D travel time matrix by Google maps API: Implementation, advantages, and implications. Ann GIS.

[R82] Wang R, Feng Z, Xue D, Liu Y, Wu R (2019). Exploring the links between population density, lifestyle, and being overweight: Secondary data analyses of middle-aged and older Chinese adults. Health and Quality of Life Outcomes.

[R83] Wang X, Rodriguez DA, Sarmiento OL, Guaje O (2019). Commute patterns and mental health: evidence from eleven latin american cities. Journal of Transport Health.

[R84] Warren TY, Barry V, Hooker SP, Sui X, Church TS, Blair SN (2011). Sedentary behaviors increase risk of cardiovascular disease mortality in men. Medicine and Science in Sports and Exercise.

[R85] Webber L, Kilpi F, Marsh T, Rtveladze K, Brown M, McPherson K (2012). High rates of obesity and non-communicable diseases predicted across Latin America. PLoS ONE.

[R86] Weichenthal S, Hoppin JA, Reeves F (2014). Obesity and the cardiovascular health effects of fine particulate air pollution. Obesity.

[R87] Werneck AO, Baldew SS, Miranda JJ, Díaz Arnesto O, Stubbs B, Silva DR (2019). Physical activity and sedentary behavior patterns and sociodemographic correlates in 116,982 adults from six South American countries: The South American physical activity and sedentary behavior network (SAPASEN). International Journal of Behavioral Nutrition and Physical Activity.

[R88] World Health Organization (WHO), n.d.World Health Organization (WHO) Body Mass Index-BMI.

[R89] Wu H (2018). 2018 Transport findings.

[R90] Yin C, Yao X, Sun B (2022). Population density and obesity in rural China: Mediation effects of car ownership. Transportation Research Part D: Transport and Environment.

[R91] Zhao Z, Kaestner R (2010). Effects of urban sprawl on obesity. Journal of Health Economics.

