# Supplementary material for: Is city-level travel time by car associated with individual obesity or diabetes in Latin American cities? Evidence from 178 cities in the SALURBAL project
Source: Cities. Author manuscript; Available in PMC 2022 Dec 1. (PMC7613723; doi:10.1016/j.cities.2022.103899)
Supplement: Supplementary Data [file EMS155489-supplement-Supplementary_Data.docx]

| **Table S1.** Description of national health surveys used in the study. | | |
| --- | --- | --- |
| **Country (year)** | **Survey** | **Description** |
| Brazil (2013) | *Pesquisa Nacional de Saúde, PNS 2013* | Total sample: 64,308 adults 18+ years, Sampling: Multistage [census tracts or groups of census tracts; households; per-son 18 years or older], Stratified [capital city, metropolitan region, or integrated economic development region, then rest of municipalities; Urban/rural; total household income], Method: CAPI, Response rate: 91.9%, Geographic coverage: Regions (5) States or federation units (27), state capitals (27), Representation: Regions (5) States or federation units (27), state capitals (27), urban and rural, metropolitan areas and development integrated areas. |
| Chile (2010) | *Encuesta Nacional de Salud, ENS 2010* | Total sample: 5,293 individuals ≥15 years, Sampling: Multistage [Comunas; Segments within comunas; household; per-son 15 years or older], Stratified [urban/rural with three groups of population sizes], Method: CAPI, Response rate: 85%, Geographic coverage: National. Representation: National, Regions (15), urban/rural. |
| Colombia (2007) | *Encuesta Nacional de Salud, ENS 2007* | Total sample: 102,677 (41,281 adults 18-69 years), Sampling: Multistage [Municipalities or combination of municipalities if small; Manzanas; household; person adults 18-69 and all children 17 and under], Stratified [region; urbanization of municipal seats; urban/rural municipal, population; unsatisfied basic needs], Method: CAPI, Response rate: 81%, Geographic coverage: National. Representation: Region, department, sub-region, urban area of municipal capitals, urban/rural, by poverty level. |
| Mexico (2012) | *Encuesta Nacional de Salud y Nutrición, ENSANUT 2012* | Total sample: 46,277 adults 18+ years, Sampling: Multistage [AGEB; Manzana (urban) or pseudo-manzanas within localidades (rural); Households; 1 person within each of the groups (0-4 years, 5-9 years, 10-19 years, 20 years and older, recent medical ser-vice user)], Stratified [socioeconomic status of AGEB at the state level], Method: CAPI, Response rate: 87%, Geographic coverage: National. Representation: National, state, metropolitan areas, urban/rural, high/low SES. |
| Peru (2016) | *Encuesta Nacional de Demografia y Salud, ENDES2016* | Total sample: 122,368 (adults 18+ years N=32158), Sampling: Multistage [Conglomerado (set of census blocks – urban) or Empadronamiento (set of households – rural); Households; One person within each of the groups (>15 years, females 15-49 years, children <5 years, children <12 years)], Stratified [Department; Urban/Rural], Method: CAPI, Response rate: 98.2%, Geographic coverage: National. Representation: National, Urban National, Rural National, Natural Region: Lima Metropolitan area, coast/mountain/jungle. |

**Figure S1**. Sample flowchart.


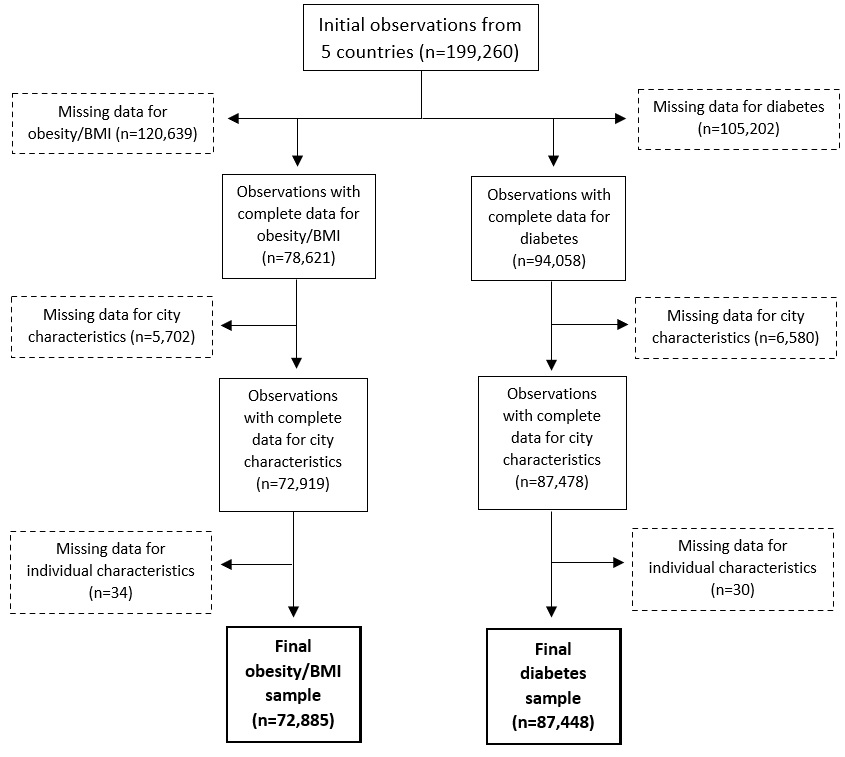


**Figure S2**. Directed Acyclic Graph (DAG).


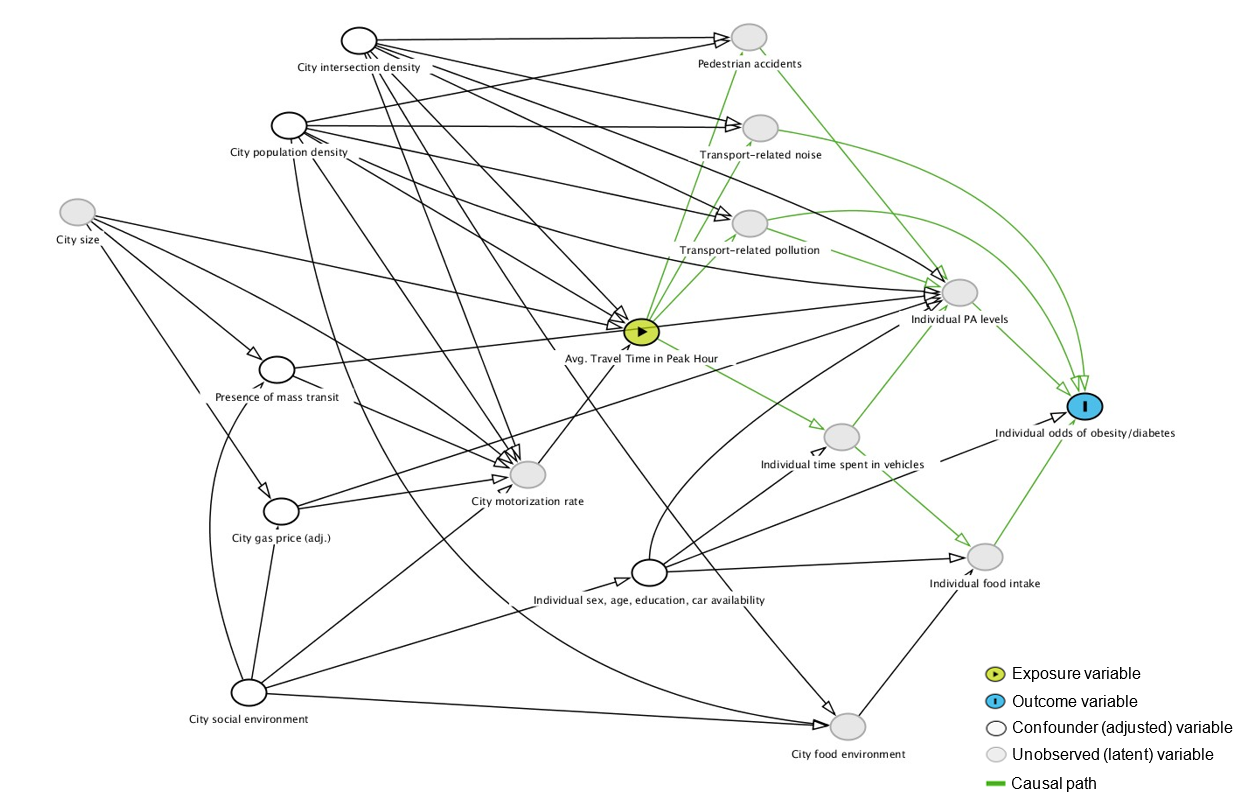


| **Table S2**. Characteristics of the excluded and included observations for the obesity and diabetes samples. | | | | |
| --- | --- | --- | --- | --- |
|  | **Obesity** | | **Diabetes** | |
|  | **Excluded (n=165,745)** | **Included (n=72,885)** | **Excluded (n=151,182)** | **Included (n=87,448)** |
| **Individual characteristics** |  |  |  |  |
| Sex |  |  |  |  |
| Female | 88,699 (53.5) | 42,276 (58.0) | 79,799 (52.8) | 5,1176 (58.5) |
| Male | 77,046 (46.5) | 30,609 (42.0) | 71,383 (47.2) | 36,272 (41.5) |
| Age | 39 [27,52] | 39 [29,53] | 38 [27,52] | 40 [30,53] |
| Education |  |  |  |  |
| < Primary | 29,359 (17.7) | 14,684 (20.2) | 26,605 (17.6) | 17,438 (19.9) |
| Primary | 59,868 (36.1) | 21,963 (30.1) | 55,095 (36.4) | 26,736 (30.6) |
| Secondary | 55,321 (33.4) | 27,345 (37.5) | 50,192 (33.2) | 32,474 (37.1) |
| University | 20,804 (12.6) | 8,893 (12.2) | 18,897 (12.5) | 10,800 (12.4) |
| Household car ownership |  |  |  |  |
| No | 77,215 (61.1) | 48,937 (67.1) | 65,180 (58.3) | 60,972 (69.7) |
| Yes | 49,053 (38.8) | 23,948 (32.9) | 46,525 (41.6) | 26,476 (30.3) |
| **City characteristics** |  |  |  |  |
| Peak-hour travel time (min.) | 22.5 [16.6,33.3] | 23.3 [16.7,33.3] | 24.0 [16.9,33.3] | 22.2 [15.0,32.5] |
| Population density (pop. / ha) | 74.6 [58.9, 97.9] | 84.4 [63.1, 113.4] | 73.5 [57.3, 88.3] | 91.8 [66.3, 141.6] |
| Intersection density (n / km^2^) | 84.5 [70.1,102.5] | 87.7 [77.4,111.7] | 82.7 [70.1,96.2] | 98.7 [79.3,122.7] |
| Adjusted gas price (% of monthly min. wage) | 3.55 [2.60,6.52] | 3.56 [3.36,3.97] | 3.59 [2.81,6.59] | 3.54 [2.74,3.97] |
| Presence of mass transit |  |  |  |  |
| No | 87,927 (53.1) | 37,270 (51.1) | 80,250 (53.1) | 44,947 (51.4) |
| Yes | 77,650 (46.9) | 35,615 (48.9) | 70,764 (46.9) | 42,501 (48.6) |
| Social Environment Index (z-score) | 0.32 [-0.17,0.46] | 0.12 [-0.32,0.46] | 0.28 [-0.19,0.46] | 0.19 [-0.24,0.53] |
| Values are show as n (%) or p50 [p25, p75]. | | | | |

| **Table S3**. Obesity and diabetes diagnosis by individual and city characteristics. | | | |  | | |
| --- | --- | --- | --- | --- | --- | --- |
|  | **Obesity** | | **p-value ^a^** | **Diabetes** | | **p-value ^a^** |
|  | **No (n, %)** | **Yes (n, %)** |  | **No (n, %)** | **Yes (n, %)** |  |
| **Overall** | 54,730 (75.09) | 18,155 (24.91) |  | 81,822 (93.57) | 5,626 (6.43) |  |
| **Individual characteristics** |  |  |  |  |  |  |
| Sex |  |  | <0.001 |  |  | <0.001 |
| Female | 30,568 (72.31) | 11,708 (27.69) |  | 47,684 (93.18) | 3,492 (6.82) |  |
| Male | 24,162 (78.94) | 6,447 (21.06) |  | 34,138 (94.12) | 2,134 (5.88) |  |
| Age |  |  | <0.001 |  |  | <0.001 |
| 18-29 | 16,273 (85.30) | 2,805 (14.70) |  | 21,535 (99.22) | 170 (0.78) |  |
| 30-59 | 29,946 (71.21) | 12,107 (28.79) |  | 48,662 (94.64) | 2,758 (5.36) |  |
| 60 or older | 8,511 (72.41) | 3,243 (27.59) |  | 11,625 (81.16) | 2,698 (18.84) |  |
| Education |  |  | <0.001 |  |  | <0.001 |
| < Primary | 10,459 (71.23) | 4,225 (28.77) |  | 15,124 (86.73) | 2,314 (13.27) |  |
| Primary | 15,879 (72.30) | 6,084 (27.70) |  | 25,056 (93.72) | 1,680 (6.28) |  |
| Secondary | 21,402 (78.27) | 5,943 (21.73) |  | 31,322 (96.45) | 1,152 (3.55) |  |
| University | 6,990 (78.60) | 1,903 (21.40) |  | 10,320 (95.56) | 480 (4.44) |  |
| Household car ownership |  |  | 0.249 |  |  | 0.493 |
| No | 36,811 (75.22) | 12,126 (24.78) |  | 57,026 (93.53) | 3,946 (6.47) |  |
| Yes | 17,919 (74.82) | 6,029 (25.18) |  | 24,796 (93.65) | 1,680 (6.35) |  |
| **City characteristics** |  |  |  |  |  |  |
| Peak-hour travel time (min.) (tertiles) |  |  | <0.001 |  |  | 0.008 |
| Lowest (5.1 - 18.0) | 17,421 (72.63) | 6,566 (27.37) |  | 29,603 (93.86) | 1,935 (6.14) |  |
| Middle (18.1 - 27.5) | 17,583 (75.77) | 5,624 (24.23) |  | 25,961 (93.56) | 1,786 (6.44) |  |
| Highest (27.6 - 66.1) | 19,726 (76.78) | 5,965 (23.22) |  | 26,258 (93.24) | 1,905 (6.76) |  |
| Population density (pop. / ha) (tertiles) |  |  | <0.001 |  |  | <0.001 |
| Lowest (27.6 - 66.8) | 14,963 (71.75) | 5,890 (28.25) |  | 20,166 (91.54) | 1,863 (8.46) |  |
| Middle (66.9 - 89.4) | 15,665 (74.14) | 5,463 (25.86) |  | 20,063 (92.83) | 1,549 (7.17) |  |
| Highest (89.5 - 294.5) | 24,102 (77.99) | 6,802 (22.01) |  | 41,593 (94.95) | 2,214 (5.05) |  |
| Intersection density (n / km^2^) (tertiles) |  |  | <0.001 |  |  | <0.001 |
| Lowest (30.2 - 78.9) | 16,035 (76.14) | 5,025 (23.86) |  | 19,847 (92.99) | 1,496 (7.01) |  |
| Middle (79.0 - 98.4) | 16,662 (76.04) | 5,251 (23.96) |  | 20,295 (92.30) | 1,693 (7.70) |  |
| Highest (98.5 - 169.6) | 22,033 (73.66) | 7,879 (26.34) |  | 41,680 (94.48) | 2,437 (5.52) |  |
| Adjusted gas price (% of monthly min. wage) (tertiles) |  |  | <0.001 |  |  | <0.001 |
| Lowest (1.2 - 3.4) | 15,838 (77.35) | 4,639 (22.65) |  | 31,918 (95.06) | 1,660 (4.94) |  |
| Middle (3.5 -3.9) | 22,825 (78.11) | 6,398 (21.89) |  | 26,775 (94.21) | 1,647 (5.79) |  |
| Highest (4.0 - 10.3) | 16,067 (69.30) | 7,118 (30.70) |  | 23,129 (90.89) | 2,319 (9.11) |  |
| Presence of mass transit |  |  | <0.001 |  |  | 0.696 |
| No | 27,267 (73.16) | 10,003 (26.84) |  | 42,070 (93.60) | 2,877 (6.40) |  |
| Yes | 27,463 (77.11) | 8,152 (22.89) |  | 39,752 (93.53) | 2,749 (6.47) |  |
| Social Environment Index (z-score) (tertiles) |  |  | 0.988 |  |  | <0.001 |
| Lowest (-2.0 - -0.1) | 20,863 (75.07) | 6,930 (24.93) |  | 28,099 (93.97) | 1,803 (6.03) |  |
| Middle (0.0 - 0.4) | 17,149 (75.13) | 5,678 (24.87) |  | 24,949 (93.59) | 1,708 (6.41) |  |
| Highest (0.5 - 1.1) | 16,718 (75.09) | 5,547 (24.91) |  | 28,774 (93.15) | 2,115 (6.85) |  |
| a. Chi-square test. | | | |  | | |

| **Figure S3**. Projected probability plot for obesity relative to city-level average travel time during peak hour (min) contrasted by population density (pop./ha). |
| --- |
|  |

| **Figure S4**. Projected probability plot for diabetes relative to city-level average travel time during peak hour (min) contrasted by population density (pop./ha). |
| --- |
|  |

| **Table S4**. Odds ratios of obesity and diabetes associated with city-level peak hour travel time and individual and city-level covariates, by city size^a^. | | | |
| --- | --- | --- | --- |
|  | **Small cities** | **Medium-sized cities** | **Large cities** |
| **Obesity (n=44,041)^b^** | OR (95% CI) | OR (95% CI) | OR (95% CI) |
| Peak-hour travel time (10 min units) | 0.988 (0.789, 1.237) | 0.895 (0.687, 1.166) | 1.120 (0.929, 1.350) |
| Male (Ref.=Female) | 0.701 (0.655, 0.751)* | 0.668 (0.618, 0.723)* | 0.741 (0.692, 0.794)* |
| Age | 1.028 (1.026, 1.030)* | 1.026 (1.024, 1.029)* | 1.024 (1.021, 1.026)* |
| University (Ref.= Less than primary) | 1.028 (0.898, 1.178) | 0.760 (0.652, 0.887)* | 0.713 (0.629, 0.809)* |
| Secondary (Ref.= Less than primary) | 1.121 (1.008, 1.248)* | 0.939 (0.833, 1.059) | 0.937 (0.844, 1.039) |
| Primary (Ref.= Less than primary) | 1.373 (1.238, 1.523)* | 1.174 (1.047, 1.317)* | 1.195 (1.076, 1.328)* |
| Car ownership (Yes) | 1.242 (1.141, 1.352)* | 1.222 (1.117, 1.337)* | 1.272 (1.180, 1.372)* |
| Population density (pop./ha) | 0.993 (0.991, 0.995)* | 0.997 (0.994, 1.001) | 0.994 (0.988, 1.001) |
| Intersection density (n/sq.km) | 1.006 (1.002, 1.010)* | 1.003 (0.997, 1.009) | 1.012 (1.002, 1.021)* |
| Adjusted gas price (% of monthly min. wage) | 1.149 (1.086, 1.216)* | 1.178 (1.081, 1.282)* | 1.222 (1.090, 1.371)* |
| Presence of mass transit (Yes) | 1.020 (0.618, 1.686) | 0.945 (0.727, 1.227) | 1.044 (0.466, 2.341) |
| Social Environment Index (z-score) | 0.902 (0.792, 1.027) | 0.906 (0.723, 1.135) | 0.685 (0.452, 1.039) |
| **Diabetes (n=87,448)** |  |  |  |
| Peak-hour travel time (10 min units) | 0.931 (0.769, 1.127) | 0.899 (0.721, 1.120) | 0.966 (0.853, 1.095) |
| Male (Ref.=Female) | 0.884 (0.803, 0.973)* | 0.903 (0.808, 1.008) | 0.833 (0.754, 0.921)* |
| Age | 1.059 (1.055, 1.062)* | 1.057 (1.053, 1.061)* | 1.055 (1.051, 1.058)* |
| University (Ref.= Less than primary) | 0.929 (0.768, 1.125) | 0.545 (0.435, 0.683)* | 0.533 (0.446, 0.636)* |
| Secondary (Ref.= Less than primary) | 0.843 (0.727, 0.976)* | 0.715 (0.607, 0.842)* | 0.653 (0.570, 0.749)* |
| Primary (Ref.= Less than primary) | 1.092 (0.965, 1.234) | 0.878 (0.765, 1.009) | 0.801 (0.702, 0.914)* |
| Car ownership (Yes) | 1.043 (0.924, 1.178) | 1.018 (0.895, 1.158) | 1.067 (0.958, 1.189) |
| Population density (pop./ha) | 0.999 (0.996, 1.001) | 0.998 (0.996, 1.001) | 0.999 (0.995, 1.004) |
| Intersection density (n/sq.km) | 0.995 (0.992, 0.999)* | 0.999 (0.994, 1.004) | 1.001 (0.994, 1.007) |
| Adjusted gas price (% of monthly min. wage) | 1.113 (1.060, 1.168)* | 1.103 (1.027, 1.185)* | 1.135 (1.046, 1.231)* |
| Presence of mass transit (Yes) | 1.142 (0.773, 1.687) | 0.940 (0.754, 1.173) | 0.919 (0.474, 1.779) |
| Social Environment Index (z-score) | 1.133 (1.014, 1.266)* | 0.985 (0.816, 1.190) | 1.060 (0.797, 1.410) |
| Mixed effects logistic regressions with random effects at the city level. Outcomes: obesity and diabetes. Key exposure: city-level travel time during peak hour in 10-minute units. a. City sizes divided by built-up area tertiles: Small cities (built-up area lower than 9,541 sq. km); Medium-sized cities (built-up area between 9,541 and 24,205 sq. km); Large cities (built-up area higher than 24,205 sq. km). b. Normal weight used as reference category (BMI between 18.5–24.9 kg/m2); underweight and overweight excluded. | | | |

| **Table S5**. Odds ratios of obesity and diabetes associated with city-level peak hour travel time and individual and city-level covariates, by respondent sex. | | |
| --- | --- | --- |
|  | **Men** | **Women** |
| **Obesity (n=44,041)^a^** | OR (95% CI) | OR (95% CI) |
| Peak-hour travel time (10 min units) | 1.008 (0.906, 1.121) | 1.004 (0.915, 1.102) |
| Age | 1.025 (1.023, 1.027)* | 1.027 (1.025, 1.029)* |
| University (Ref.= Less than primary) | 1.751 (1.541, 1.990)* | 0.526 (0.475, 0.583)* |
| Secondary (Ref.= Less than primary) | 1.704 (1.533, 1.894)* | 0.739 (0.682, 0.801)* |
| Primary (Ref.= Less than primary) | 1.481 (1.335, 1.643)* | 1.156 (1.068, 1.251)* |
| Car ownership (Yes) | 1.672 (1.551, 1.804)* | 0.999 (0.939, 1.063) |
| Population density (pop./ha) | 0.995 (0.993, 0.997)* | 0.995 (0.993, 0.997)* |
| Intersection density (n/sq.km) | 1.005 (1.002, 1.009)* | 1.007 (1.004, 1.010)* |
| Adjusted gas price (% of monthly min. wage) | 1.153 (1.094, 1.215)* | 1.168 (1.117, 1.222)* |
| Presence of mass transit (Yes) | 0.864 (0.677, 1.103) | 0.952 (0.769, 1.178) |
| Social Environment Index (z-score) | 0.915 (0.804, 1.041) | 0.884 (0.793, 0.987)* |
| **Diabetes (n=87,448)** |  |  |
| Peak-hour travel time (10 min units) | 0.998 (0.931, 1.069) | 0.990 (0.927, 1.057) |
| Age | 1.062 (1.059, 1.066)* | 1.053 (1.051, 1.056)* |
| University (Ref.= Less than primary) | 0.977 (0.824, 1.157) | 0.481 (0.413, 0.561)* |
| Secondary (Ref.= Less than primary) | 1.102 (0.959, 1.266) | 0.570 (0.511, 0.636)* |
| Primary (Ref.= Less than primary) | 1.313 (1.158, 1.488)* | 0.762 (0.694, 0.837)* |
| Car ownership (Yes) | 1.129 (1.014, 1.257)* | 0.980 (0.896, 1.071) |
| Population density (pop./ha) | 0.999 (0.998, 1.001) | 0.998 (0.997, 1.000)* |
| Intersection density (n/sq.km) | 0.996 (0.993, 0.999)* | 0.998 (0.996, 1.001) |
| Adjusted gas price (% of monthly min. wage) | 1.116 (1.071, 1.164)* | 1.132 (1.090, 1.175)* |
| Presence of mass transit (Yes) | 0.900 (0.756, 1.070) | 1.098 (0.939, 1.284) |
| Social Environment Index (z-score) | 1.123 (1.013, 1.245)* | 1.087 (0.992, 1.191) |
| Mixed effects logistic regressions with random effects at the city level. Outcomes: obesity and diabetes. Key exposure: city-level travel time during peak hour in 10-minute units. a. Normal weight used as reference category (BMI between 18.5–24.9 kg/m2); underweight and overweight excluded. | | |

| **Table S6**. Odds ratios of obesity and diabetes associated with city-level peak hour travel time and individual and city-level covariates, by car ownership at the household level. | | |
| --- | --- | --- |
|  | **Household car** | **No household car** |
| **Obesity (n=44,041)^a^** | OR (95% CI) | OR (95% CI) |
| Peak-hour travel time (10 min units) | 1.022 (0.920, 1.135) | 0.991 (0.903, 1.087) |
| Male (Ref.=Female) | 1.143 (1.065, 1.227)* | 0.550 (0.522, 0.578)* |
| Age | 1.032 (1.029, 1.034)* | 1.023 (1.021, 1.025)* |
| University (Ref.= Less than primary) | 0.681 (0.599, 0.775)* | 1.020 (0.910, 1.143) |
| Secondary (Ref.= Less than primary) | 0.965 (0.853, 1.092) | 0.973 (0.903, 1.049) |
| Primary (Ref.= Less than primary) | 1.167 (1.022, 1.332)* | 1.246 (1.160, 1.337)* |
| Population density (pop./ha) | 0.996 (0.994, 0.999)* | 0.995 (0.993, 0.996)* |
| Intersection density (n/sq.km) | 1.008 (1.004, 1.012)* | 1.006 (1.003, 1.009)* |
| Adjusted gas price (% of monthly min. wage) | 1.131 (1.071, 1.194)* | 1.182 (1.131, 1.236)* |
| Presence of mass transit (Yes) | 0.898 (0.706, 1.143) | 0.948 (0.765, 1.174) |
| Social Environment Index (z-score) | 0.762 (0.660, 0.880)* | 0.943 (0.847, 1.049) |
| **Diabetes (n=87,448)** |  |  |
| Peak-hour travel time (10 min units) | 0.963 (0.885, 1.048) | 0.995 (0.929, 1.065) |
| Male (Ref.=Female) | 1.056 (0.951, 1.171) | 0.799 (0.744, 0.858)* |
| Age | 1.063 (1.059, 1.067)* | 1.054 (1.052, 1.057)* |
| University (Ref.= Less than primary) | 0.518 (0.439, 0.610)* | 0.802 (0.682, 0.945)* |
| Secondary (Ref.= Less than primary) | 0.653 (0.562, 0.760)* | 0.745 (0.672, 0.827)* |
| Primary (Ref.= Less than primary) | 0.817 (0.699, 0.954)* | 0.942 (0.865, 1.026) |
| Population density (pop./ha) | 1.000 (0.998, 1.003) | 0.998 (0.997, 1.000)* |
| Intersection density (n/sq.km) | 0.998 (0.995, 1.002) | 0.997 (0.994, 0.999)* |
| Adjusted gas price (% of monthly min. wage) | 1.131 (1.074, 1.190)* | 1.126 (1.085, 1.168)* |
| Presence of mass transit (Yes) | 1.061 (0.867, 1.299) | 0.996 (0.846, 1.172) |
| Social Environment Index (z-score) | 0.968 (0.844, 1.109) | 1.144 (1.047, 1.251)* |
| Mixed effects logistic regressions with random effects at the city level. Outcomes: obesity and diabetes. Key exposure: city-level travel time during peak hour in 10-minute units. a. Normal weight used as reference category (BMI between 18.5–24.9 kg/m2); underweight and overweight excluded. | | |

| **Table S7**. Odds ratios of obesity and diabetes associated with city-level peak hour travel time and individual and city-level covariates, by country. | | | | | |
| --- | --- | --- | --- | --- | --- |
|  | **Brazil** | **Chile^b^** | **Colombia^b,c^** | **Mexico** | **Peru^b,c^** |
| **Obesity (n=44,041)^a^** | OR (95% CI) | OR (95% CI) | OR (95% CI) | OR (95% CI) | OR (95% CI) |
| Peak-hour travel time (10 min units) | 1.048 (0.934, 1.176) | 0.812 (0.568, 1.160) | 0.983 (0.705, 1.372) | 0.910 (0.772, 1.073) | 0.747 (0.437, 1.277) |
| Male (Ref.=Female) | 0.754 (0.711, 0.799)* | 0.844 (0.669, 1.066) | 0.647 (0.545, 0.768)* | 0.629 (0.579, 0.683)* | 0.667 (0.602, 0.740)* |
| Age | 1.022 (1.020, 1.024)* | 1.023 (1.016, 1.031)* | 1.051 (1.044, 1.058)* | 1.028 (1.025, 1.031)* | 1.029 (1.025, 1.033)* |
| University (Ref.= Less than primary) | 0.677 (0.611, 0.751)* | 0.483 (0.273, 0.854)* | 0.898 (0.622, 1.296) | 1.142 (0.954, 1.367) | 1.255 (1.014, 1.552)* |
| Secondary (Ref.= Less than primary) | 0.938 (0.864, 1.019) | 0.773 (0.500, 1.194) | 0.867 (0.670, 1.122) | 1.097 (0.950, 1.267) | 1.308 (1.098, 1.557)* |
| Primary (Ref.= Less than primary) | 1.071 (0.982, 1.169) | 1.124 (0.745, 1.695) | 1.089 (0.861, 1.376) | 1.406 (1.248, 1.585)* | 1.930 (1.586, 2.350)* |
| Car ownership (Yes) | 1.324 (1.243, 1.411)* | 1.096 (0.858, 1.400) | 1.308 (0.997, 1.715) | 1.059 (0.962, 1.165) | 1.413 (1.221, 1.635)* |
| Population density (pop./ha) | 1.000 (0.996, 1.003) | 0.990 (0.982, 0.998)* | 0.997 (0.992, 1.001) | 0.995 (0.988, 1.001) | 0.995 (0.987, 1.004) |
| Intersection density (n/sq.km) | 1.004 (0.996, 1.011) | 1.013 (0.996, 1.031) | 1.002 (0.995, 1.009) | 1.004 (1.000, 1.008)* | 1.007 (0.997, 1.016) |
| Adjusted gas price (% of monthly min. wage) | 1.135 (0.770, 1.675) | 0.979 (0.211, 4.547) | 1.337 (0.619, 2.889) | 0.427 (0.173, 1.053) | 0.587 (0.218, 1.577) |
| Presence of mass transit (Yes) | 0.985 (0.806, 1.203) | 1.181 (0.565, 2.469) | 1.165 (0.616, 2.205) | 1.096 (0.805, 1.493) | 4.875 (0.413, 57.578) |
| Social Environment Index (z-score) | 0.893 (0.738, 1.081) | 1.031 (0.473, 2.250) | 0.907 (0.686, 1.199) | 0.965 (0.814, 1.144) | 1.084 (0.633, 1.857) |
| **Diabetes (n=87,448)** |  |  |  |  |  |
| Peak-hour travel time (10 min units) | 1.015 (0.922, 1.116) | 0.815 (0.523, 1.269) | 1.030 (0.968, 1.096) | 1.036 (0.900, 1.192) | 1.017 (0.970, 1.065) |
| Male (Ref.=Female) | 0.896 (0.817, 0.982)* | 0.939 (0.702, 1.255) | 0.815 (0.696, 0.955)* | 0.844 (0.762, 0.936)* | 0.871 (0.715, 1.060) |
| Age | 1.056 (1.053, 1.059)* | 1.054 (1.044, 1.064)* | 1.057 (1.051, 1.064)* | 1.055 (1.051, 1.058)* | 1.066 (1.060, 1.073)* |
| University (Ref.= Less than primary) | 0.543 (0.465, 0.635)* | 0.827 (0.423, 1.619) | 0.819 (0.575, 1.167) | 0.590 (0.466, 0.746)* | 1.811 (1.276, 2.571)* |
| Secondary (Ref.= Less than primary) | 0.631 (0.558, 0.714)* | 0.694 (0.433, 1.114) | 0.901 (0.711, 1.142) | 0.697 (0.584, 0.833)* | 1.726 (1.297, 2.296)* |
| Primary (Ref.= Less than primary) | 0.831 (0.733, 0.942)* | 1.214 (0.821, 1.795) | 1.180 (0.971, 1.433) | 0.816 (0.720, 0.924)* | 1.820 (1.304, 2.540)* |
| Car ownership (Yes) | 1.041 (0.944, 1.148) | 1.348 (0.989, 1.839) | 1.021 (0.798, 1.306) | 1.019 (0.901, 1.153) | 1.226 (0.953, 1.577) |
| Population density (pop./ha) | 0.999 (0.996, 1.003) | 0.995 (0.985, 1.005) |  | 0.997 (0.991, 1.003) |  |
| Intersection density (n/sq.km) | 1.006 (0.999, 1.013) | 1.020 (0.999, 1.042) |  | 0.998 (0.995, 1.001) |  |
| Adjusted gas price (% of monthly min. wage) | 0.828 (0.597, 1.149) | 1.883 (0.270, 13.126) |  | 0.446 (0.198, 1.005) |  |
| Presence of mass transit (Yes) | 0.920 (0.769, 1.100) | 1.521 (0.614, 3.767) |  | 1.034 (0.798, 1.341) |  |
| Social Environment Index (z-score) | 1.110 (0.934, 1.320) | 0.905 (0.338, 2.427) |  | 1.024 (0.881, 1.190) |  |
| Mixed effects logistic regressions with random effects at the city level. Outcomes: obesity and diabetes. Key exposure: city-level travel time during peak hour in 10-minute units. a. Normal weight used as reference category (BMI between 18.5–24.9 kg/m2); underweight and overweight excluded. b. For the obesity sample from Chile and the diabetes sample from Chile, Colombia and Peru, random effect for city was not included due to lack of between-city variance after adjustment for city-level factors. c. For the diabetes sample from Colombia and Peru, models excluded city-level covariates due to lack of model convergence. | | | | | |

| **Table S8.** Odds ratios of obesity and diabetes associated with city-level peak hour travel time and individual and city-level covariates, adjusting by countries as fixed effects. | |
| --- | --- |
| **Obesity (n=44,041)^b^** | **OR (95% CI)** |
| Peak-hour travel time (10 min units) | 1.012 (0.929, 1.103) |
| Male (Ref.=Female) | 0.707 (0.678, 0.737)* |
| Age | 1.026 (1.024, 1.027)* |
| University (Ref.= Less than primary) | 0.816 (0.754, 0.883)* |
| Secondary (Ref.= Less than primary) | 0.991 (0.931, 1.056) |
| Primary (Ref.= Less than primary) | 1.244 (1.169, 1.323)* |
| Car ownership (Yes) | 1.248 (1.190, 1.309)* |
| Population density (pop./ha) | 0.997 (0.994, 0.999)* |
| Intersection density (n/sq.km) | 1.003 (1.001, 1.006)* |
| Adjusted gas price (% of monthly min. wage) | 0.945 (0.700, 1.274) |
| Presence of mass transit (Yes) | 1.011 (0.846, 1.208) |
| Social Environment Index (z-score) | 0.914 (0.823, 1.015) |
| Chile (Ref.= Brazil) | 1.552 (1.007, 2.391) |
| Colombia (Ref.= Brazil) | 0.739 (0.462, 1.182) |
| Mexico (Ref.= Brazil) | 2.499 (0.978, 6.389) |
| Peru (Ref.= Brazil) | 1.546 (1.207, 1.979)* |
| **Diabetes (n=87,448)** |  |
| Peak-hour travel time (10 min units) | 1.023 (0.960, 1.090) |
| Male (Ref.=Female) | 0.873 (0.824, 0.926)* |
| Age | 1.057 (1.055, 1.059)* |
| University (Ref.= Less than primary) | 0.647 (0.578, 0.724)* |
| Secondary (Ref.= Less than primary) | 0.734 (0.674, 0.800)* |
| Primary (Ref.= Less than primary) | 0.916 (0.849, 0.987)* |
| Car ownership (Yes) | 1.041 (0.972, 1.115) |
| Population density (pop./ha) | 0.999 (0.997, 1.001) |
| Intersection density (n/sq.km) | 0.998 (0.996, 1.000) |
| Adjusted gas price (% of monthly min. wage) | 0.810 (0.633, 1.037) |
| Presence of mass transit (Yes) | 0.988 (0.863, 1.131) |
| Social Environment Index (z-score) | 1.021 (0.937, 1.114) |
| Chile (Ref.= Brazil) | 0.929 (0.646, 1.337) |
| Colombia (Ref.= Brazil) | 0.720 (0.495, 1.048) |
| Mexico (Ref.= Brazil) | 2.941 (1.354, 6.388)* |
| Peru (Ref.= Brazil) | 0.682 (0.551, 0.843)* |
| Mixed effects logistic regressions with random effects at the city level. Outcomes: obesity and diabetes. Key exposure: city-level travel time during peak hour in 10-minute units. a. Normal weight used as reference category (BMI between 18.5–24.9 kg/m2); underweight and overweight excluded. | |

| **Table S9**. Sensitivity analyses for weight status categorization. | | | | | |
| --- | --- | --- | --- | --- | --- |
|  | BMI^a^ | Overweight+Obesity^b^ | BMI categories^c^ | | |
|  |  |  | *Underweight* | *Overweight* | *Obesity* |
|  | **Coef. (95% CI)** | **OR (95% CI)** | **OR (95% CI)** | **OR (95% CI)** | **OR (95% CI)** |
| Peak-hour travel time (10 min units) | 0.013 (-0.175, 0.201) | 1.009 (0.944, 1.078) | 0.879 (0.793, 0.974)* | 1.024 (0.972, 1.079) | 1.012 (0.934, 1.097) |
| Male (Ref.=Female) | -0.695 (-0.773, -0.618)* | 0.934 (0.904, 0.964)* | 0.772 (0.695, 0.859)* | 1.097 (1.060, 1.136)* | 0.713 (0.685, 0.742)* |
| Age | 0.053 (0.050, 0.056)* | 1.025 (1.024, 1.026)* | 0.983 (0.979, 0.987)* | 1.023 (1.022, 1.024)* | 1.027 (1.025, 1.028)* |
| University (Ref.= Less than primary) | -0.215 (-0.367, -0.064)* | 0.994 (0.934, 1.057) | 0.476 (0.378, 0.600)* | 1.118 (1.045, 1.196)* | 0.831 (0.769, 0.898)* |
| Secondary (Ref.= Less than primary) | 0.069 (-0.052, 0.191) | 1.139 (1.083, 1.197)* | 0.676 (0.579, 0.790)* | 1.243 (1.177, 1.314)* | 1.010 (0.950, 1.074) |
| Primary (Ref.= Less than primary) | 0.473 (0.353, 0.593)* | 1.263 (1.202, 1.328)* | 0.781 (0.670, 0.911)* | 1.282 (1.213, 1.355)* | 1.251 (1.178, 1.329)* |
| Car ownership (Yes) | 0.470 (0.379, 0.562)* | 1.196 (1.152, 1.242)* | 0.685 (0.599, 0.784)* | 1.169 (1.121, 1.218)* | 1.237 (1.181, 1.296)* |
| Population density (pop./ha) | -0.010 (-0.014, -0.007)* | 0.996 (0.995, 0.998)* | 1.002 (0.999, 1.004) | 0.998 (0.997, 0.999)* | 0.995 (0.993, 0.996)* |
| Intersection density (n/sq.km) | 0.012 (0.006, 0.017)* | 1.004 (1.002, 1.006)* | 0.999 (0.995, 1.003) | 1.003 (1.001, 1.005)* | 1.006 (1.004, 1.009)* |
| Adjusted gas price (% of monthly min. wage) | 0.297 (0.210, 0.384)* | 1.105 (1.070, 1.141)* | 1.017 (0.958, 1.080) | 1.077 (1.049, 1.106)* | 1.159 (1.116, 1.204)* |
| Presence of mass transit (Yes) | -0.302 (-0.732, 0.129) | 0.913 (0.784, 1.063) | 1.382 (1.088, 1.757)* | 0.907 (0.804, 1.024) | 0.918 (0.764, 1.104) |
| Social Environment Index (z-score) | -0.143 (-0.354, 0.069) | 0.905 (0.838, 0.977)* | 1.065 (0.926, 1.224) | 0.897 (0.841, 0.956)* | 0.907 (0.826, 0.995)* |
| a. Mixed effects linear regression with Body Mass Index (BMI) as the outcome. N=72,885. b. Mixed effects logistic regression with Overweight+Obesity as the outcome. Normal weight used as reference category (BMI between 18.5–24.9 kg/m2). Underweight excluded. N=71,320. c. Multinomial logistic regression with BMI World Health Organization categories as the outcome. Normal weight used as reference category (BMI between 18.5–24.9 kg/m2). N=72,885. *p-value < 0.05 | | | | | |
